# Immunolocalization and phylogenetic profiling of the feather protein with the highest cysteine content

**DOI:** 10.1007/s00709-019-01381-3

**Published:** 2019-04-29

**Authors:** Julia Lachner, Florian Ehrlich, Veronika Mlitz, Marcela Hermann, Lorenzo Alibardi, Erwin Tschachler, Leopold Eckhart

**Affiliations:** 1 Research Division of Biology and Pathobiology of the Skin, Department of Dermatology, Medical University of Vienna, Vienna, Austria; 2 Department of Medical Biochemistry, Medical University of Vienna, Vienna, Austria; 3 Comparative Histolab, Padova, Italy

**Keywords:** Feather, Scale, Epidermis, Differentiation, Bird, Crocodile

## Abstract

Feathers are the most complex skin appendages of vertebrates. Mature feathers consist of interconnected dead keratinocytes that are filled with heavily cross-linked proteins. Although the molecular architecture determines essential functions of feathers, only few feather proteins have been characterized with regard to their amino acid sequences and evolution. Here, we identify Epidermal Differentiation protein containing DPCC Motifs (EDDM) as a cysteine-rich protein that has co-evolved with other feather proteins. The *EDDM* gene is located within the avian epidermal differentiation complex (EDC), a cluster of genes that has originated and diversified in amniotes. *EDDM* shares the exon-intron organization with EDC genes of other amniotes, including humans, and a gene encoding an EDDM-like protein is present in crocodilians, suggesting that avian *EDDM* arose by sequence modification of an epidermal differentiation gene present in a common ancestor of archosaurs. The EDDM protein contains multiple sequence repeats and a higher number of cysteine residues than any other protein encoded in the EDC. Immunohistochemical analysis of chicken skin and skin appendages showed expression of EDDM in barb and barbules of feathers as well as in the subperiderm on embryonic scutate scales. These results suggest that the diversification and differential expression of *EDDM*, besides other EDC genes, was instrumental in facilitating the evolution of the most complex molecular architecture of feathers.

**Electronic supplementary material:**

The online version of this article (10.1007/s00709-019-01381-3) contains supplementary material, which is available to authorized users.

## Introduction

Feathers are the characteristic for birds and more complex than any other skin appendages of vertebrates. They are also diverse in shape in order to accomplish different functions, including thermal insulation, communication, and flight. Pennaceous feathers, i.e., the main type of feathers in adult birds, are comprised of a shaft (rachis) and two levels of branches that are called barbs and barbules. This hierarchical organization is established in a process of differential growth, cell death, and cornification of epithelial cells, as described in extensive reviews (Chuong 1993; Prum and Dyck 2003; Prum 2005; Maderson et al. 2009; Chen et al. 2015; Alibardi 2017).

The evolutionary origin of feathers has been traced back to extinct members of the clade Dinosauria. The only extant representatives of Dinosauria are the birds and their closest modern relatives are crocodilians. Various models for the modification of ancestral development and differentiation programs of skin cells have been proposed to explain the evolution of feathers (Yu et al. 2002; Ng et al. 2015; Wu et al. 2015; Mlitz et al. 2014). Importantly, feather barbs and barbules and the subperiderm, a layer of the embryonic epidermis present not only in birds but also in crocodilians, share the same differentiation markers. Together with topological considerations, this shared gene expression signature has led to the hypothesis that the evolution of feather growth and regeneration depended on the modification of an embryonic development program of archosaurian skin for new purposes (Sawyer and Knapp 2003; Sawyer et al. 2005; Strasser et al. 2015; Alibardi et al. 2016), thus representing an example of evolutionary co-option (True and Carroll 2002; Prum 2005).

The mechanical stability and stress resistance of feathers are facilitated by their microarchitecture and biochemical composition. Besides a minor content of lipids, proteins constitute more than 90% of the feather mass (Bolliger and Varga 1961). The major protein components of feathers are Corneous Beta Proteins (CBPs), traditionally referred to as beta-keratins (Gregg and Rogers 1986; Fraser and Parry 2008; Greenwold and Sawyer 2011, 2013; Greenwold et al. 2014; Alibardi 2017; Holthaus et al. 2018a). CBPs are not related to keratin intermediate filament proteins (Eckhart and Ehrlich 2018; Holthaus et al. 2018a) but to proteins encoded in the Epidermal Differentiation Complex (EDC), a cluster of genes for protein components of cornifying keratinocytes of amniotes (Strasser et al. 2014). Many CBP genes are located in a sub-cluster of the EDC (Strasser et al. 2014). Recent reports have, however, shown that, besides CBPs, other EDC-encoded proteins and also intermediate filament keratins which are encoded by genes outside the EDC are present in different regions of feathers (Ng et al. 2014; Ng et al. 2015; Wu et al. 2015; Alibardi 2013). Most notably epidermal differentiation cysteine-rich protein (EDCRP) (Strasser et al. 2015) and epidermal differentiation proteins starting with MTF motif, Met-Thre-Phe, and rich in Histidine (EDMTFH) (Alibardi et al. 2016) are important feather components. While the mechanism by which EDMTFH may contribute to feather stabilization is unclear, EDCRP is likely to form multiple intermolecular disulfide bonds and thereby to enhance protein cross-linking in feathers (Strasser et al. 2015). The analysis of proteome data of chicken skin appendages has suggested that further proteins encoded by EDC genes are present in feathers (Rice et al. 2013; Strasser et al. 2014). Epidermal Differentiation protein containing DPCC (Asp-Pro-Cys-Cys) amino acid Motifs (EDDM) was one of these EDC proteins abundant in the proteome of chicken feathers but its expression pattern in feather cells and the possible role of EDDM in the evolution of feathers have remained unknown.

The aims of the present study were to determine the expression pattern of EDDM in chicken tissues, to identify avian and non-avian orthologs of EDDM and to develop a model for the evolutionary history of the *EDDM* gene.

## Materials and methods

### Comparative genomics and sequence analysis

Using the amino acid sequence of chicken (*Gallus gallus*) EDDM protein as query in tBLASTn searches (Altschul et al. 1990) at the NCBI GenBank website (http://www.ncbi.nlm.nih.gov/), *EDDM* orthologs were identified in the genome sequences of duck (*Anas platyrhynchos*), pigeon (*Columba livia*), falcon (*Falco cherrug*), Adélie penguin (*Pygoscelis adeliae*), emperor penguin (*Aptenodytes forsteri*), loon (*Gavia stellata*), flycatcher (*Ficedula albicollis*), canary (*Serinus canaria*), cuckoo roller (*Leptosomus discolor*), ostrich (*Struthio camelus*), greater rhea (*Pterocnemia pennata*), and great spotted kiwi (*Apteryx haastii*) (Jarvis et al. 2014); sequences of crocodilian *EDDML* were identified in the genome sequences of the American alligator (*Alligator mississippiensis*), the gharial (*Gavialis gangeticus*), and the saltwater crocodile (*Crocodylus porosus*) (Green et al. 2014; Holthaus et al. 2018b) (Suppl. Table S1). Amino acid sequences were obtained by translation of the coding region present in exon 2 of these genes. The amino acid sequences were aligned using Multalin (http://multalin.toulouse.inra.fr/multalin/) (Corpet 1988) with manual adjustment. Sequence logos were generated online with the Weblogo software (Crooks et al. 2004). Orthologs of the non-coding exon 1 of chicken *EDDM* were identified by BLASTn search of nucleotide sequences on the 5′-side of *EDDM* and *EDDML* genes.

### Preparation of chicken tissues

Chickens were maintained and eggs were incubated according to published protocols (Eresheim et al. 2014). At Hamburger-Hamilton (HH) stages 35, 39, and 44 (Hamburger and Hamilton 1992), tissues were prepared from chicken embryos that were euthanized by decapitation. The tissue samples were fixed with 7.5% formaldehyde and embedded in paraffin as described previously (Mlitz et al. 2014). RNA was prepared with the Trifast reagent (VWR) according to a published protocol (Mlitz et al. 2014).

### Quantitative reverse-transcription polymerase chain reaction

RNA was reverse-transcribed to cDNA with iScript cDNA Synthesis Kit (Bio-Rad, Hercules, CA) and the quantitative PCR was performed using the Lightcycler 480 DNA SYBR Green I master kit on a Roche LightCycler® (LC480) according to the manufacturer’s protocol. *EDDM* mRNA was amplified with the intron-spanning primer pair EDDM-f (5′-CGGCATTACTCCATCAGCTG-3′) and EDDM-r (5′-AACATCGGAGGGCTCAAGAA-3′). As a control transcript, *Casp3* mRNA was amplified with primers reported previously (Strasser et al. 2015).

### Generation of an antibody against EDDM

The peptide CYYARVPQGTTTYLKL, corresponding to amino acid residues 49–64 of chicken EDDM (Suppl. Fig. S1) was synthesized and coupled to keyhole limpet hemocyanin (KLH) by GeneCust, Ellange, Luxembourg. Six injections of 100 μg KLH-coupled peptide were performed to generate an antiserum in mice according to a published protocol (Eckhart et al. 2008).

### Immunohistochemistry

Immunohistochemical stainings were performed according to published protocols (Mlitz et al. 2014; Alibardi et al. 2016). In brief, tissues were sectioned at 4-μm thickness and antigens were demasked with citrate buffer, pH 6 (Dako). Endogenous peroxidase was blocked with hydrogen peroxide. Mouse anti-EDDM antiserum at a dilution of 1:200 was used as primary antibody. Biotinylated sheep anti-mouse immunoglobulin (RPN1001V, lot 9793564, GE Healthcare, Chalfont, UK) at a dilution of 1:200 was used as secondary antibody, and sheep serum (10%) was added to prevent unspecific binding. In control experiments, the primary antibody was replaced with preimmune serum. The samples were incubated with streptavidin-biotin-horseradish peroxidase (HRP) complex and 3-amino-9-ethylcarbazole (DakoCytomation, Glostrup, Denmark) to develop red color. Nuclei were counterstained with hematoxylin.

## Results

### The *EDDM* gene encodes the protein with the highest number of cysteine residues among chicken EDC proteins

**Fig. 1:**
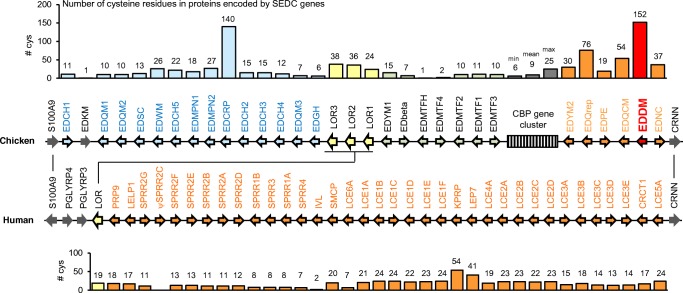
*EDDM* is located in the EDC and encodes a cysteine-rich protein. The EDC gene locus of humans (chromosome 1q21) and chicken (chromosome 25) is schematically depicted. The orientation of the genes is indicated by arrows; orthologs are connected by vertical lines. Colors distinguish genes within different domains of the EDC. The position of the corneous beta protein (CBP)/beta-keratin gene cluster is indicated by a striped box in the chicken EDC. The number of cysteine residues in proteins encoded by Single-coding exon EDC (SEDC) genes is shown in diagrams above the chicken and below the human EDC schematics. Values above bars indicate the number of cysteine residues in the proteins. The bars above the CBP cluster indicate the minimum (min), mean, and maximum (max) number of cysteine residues in CBPs

The *EDDM* gene is located in the EDC and has two exons. As the entire coding sequence is located in exon 2, it belongs to the Single-coding exon EDC (SEDC) genes (Strasser et al. 2014), which are located on the 5′-side of the evolutionarily conserved *Cornulin* (*Crnn*) gene in both chicken and human (Fig. 1). *EDDM* does not have an ortholog in humans. Cysteine residues that allow cross-linking via disulfide bonds are present in different numbers in SEDC proteins both in chicken and humans. EDCRP has 140 cysteine residues in a total of 385 amino acid residues which represents the highest relative content of cysteine (36%) among chicken EDC proteins (Strasser et al. 2015). However, the highest absolute number cysteine residues (*n* = 152) is present in EDDM (total number amino acid residues, *n* = 657) (Fig. 1; Suppl. Table S1), suggesting that this protein is capable of serving a role as a cross-linkable structural protein.

### EDDM is expressed in feather barbs and in the subperiderm

**Fig. 2:**
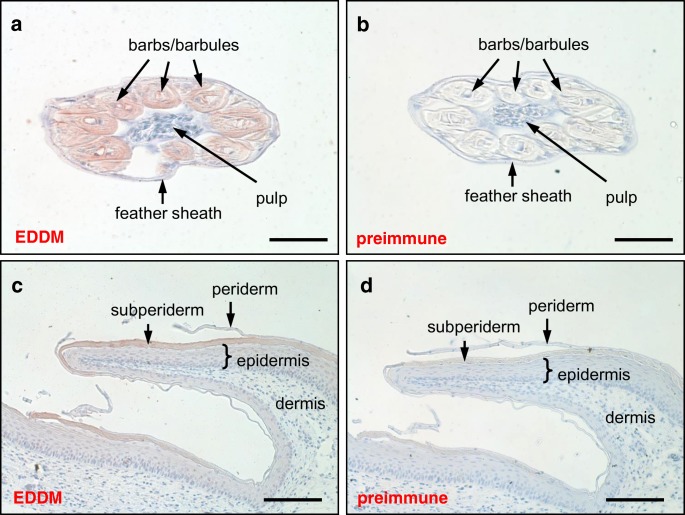
Immunohistochemial analysis of EDDM in the chicken. EDDM was detected by immunohistochemistry (red) in chicken feathers (**a**) and scutate scales (**c**) at stage HH44. In control experiments, the primary antibody was replaced by the preimmune serum (**b**, **d**). Nuclei were counterstained with hematoxylin (blue). Bars: 50 μm (**a**, **b**), 100 μm (**c**, **d**)

To determine the distribution of EDDM in chicken tissues, we generated a mouse antiserum against a unique internal peptide of EDDM (Suppl. Fig. S1), and used this antibody for immunohistochemical studies. The studies of gene expression were carried out on embryonic tissues to compare different growth stages of skin appendages and to investigate embryo-specific epithelial cells of the periderm and subperiderm. Feather buds at development stages HH35 (Suppl. Fig. S2a) and HH39 (Suppl. Fig. S2b) and the pulp and sheath of feathers at HH44 (Fig. 2a) were immunonegative. By contrast, EDDM was detected at highest signal intensity in barbs and barbules of feathers (Fig. 2a; Suppl. Fig. S2c). EDDM was also detected in the subperiderm layer on scutate scales at stage HH44 (Fig. 2c). The staining intensities in the epidermis, the periderm (Fig. 2c), and the epithelium of the tongue (Suppl. Fig. S2e) were low or absent. Negative control stainings in which the EDDM antiserum was replaced by preimmune serum (Fig. 2b, d; Suppl. Fig. S2d) showed no signals in feathers and subperiderm. The intensity of immunostaining of EDDM protein correlated with EDDM mRNA abundance in feathers during embryonic development (Suppl. Fig. S2f). In summary, immunohistochemical and RT-PCR analysis demonstrated that EDDM is abundantly expressed in feathers and, at lower levels, in the subperiderm of embryonic scutate scales.

### Avian EDDM and crocodilian EDDM-like proteins contain multiple cysteine-rich sequence repeats

**Fig. 3:**
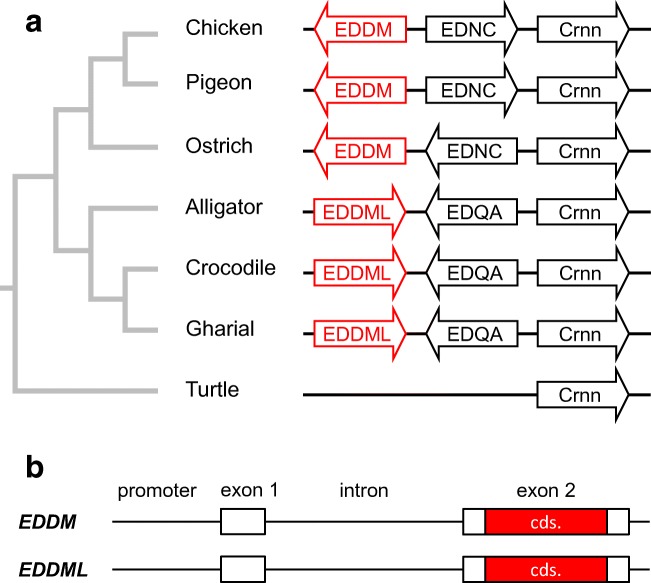
An *EDDM-like* (*EDDML*) gene is present in crocodilians. (**a**) Schematic depiction of the EDDM gene locus in birds and homologous loci in crocodilians and turtle. Species: chicken (*Gallus gallus*), pigeon (*Columba livia*), ostrich (*Struthio camelus*), American alligator (*Alligator mississippiensis*), saltwater crocodile (*Crocodylus porosus*), gharial (*Gavialis gangeticus*), turtle (*Chrysemys picta*). (**b**) Schematic organization of *EDDM* and *EDDML* genes. cds., coding sequence

To determine conserved and variable parts of the EDDM protein, we identified EDDM orthologs in genome sequences of vertebrates and compared nucleotide sequences of the genes and amino acid sequences of the encoded proteins. *EDDM* is conserved among birds and an *EDDM-like* (*EDDML*) gene is present in crocodilians but not in any other species of vertebrates investigated (Fig. 3a). In addition to the *EDDML* genes of the American alligator and the saltwater crocodile reported previously (Holthaus et al. 2018b), we could also identify *EDDML* of the gharial (Suppl. Table S1; Fig. 3a). The non-coding exon 1 and the proximal promoter was identified in *EDDM* genes of birds and in *EDDML* genes of crocodilians (Fig. 3b; Suppl. Fig. S3). Interestingly, the promoters of avian *EDDM* genes contained a canonical TATA box, whereas this important element of transcriptional control was modified in sequence in the promoters of crocodilian *EDDML* genes (Suppl. Fig. S3).

**Fig. 4:**
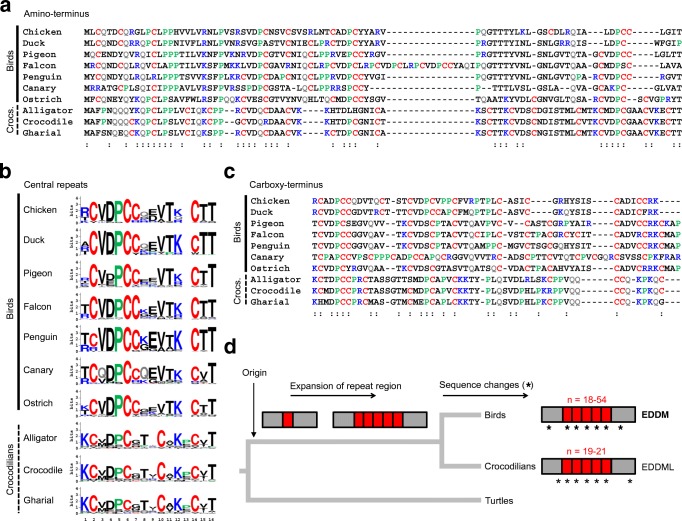
Amino acid sequence features and evolution of EDDM. The amino acid sequences of the amino-terminus (**a**), the central repeats (**b**), and the carboxy-terminus (**c**) of avian EDDM and crocodilian EDDML proteins were aligned. Colored fonts indicate the amino acid residues C, K, P, Q, and R. The symbol “:” below the sequence alignments in **a** and **c** mark positions of amino acid residues that are conserved in representatives of both EDDM and EDDML. (**b**) The conservation of residues in the sequence repeats in the central region of EDDM and EDDML is indicated by sequence logos. Species: chicken (*Gallus gallus*), duck (*Anas platyrhynchos*), pigeon (*Columba livia*), falcon (*Falco cherrug*), penguin (*Pygoscelis adeliae*), canary (*Serinus canaria*), ostrich (*Struthio camelus*), American alligator (*Alligator mississippiensis*), saltwater crocodile (*Crocodylus porosus*), gharial (*Gavialis gangeticus*). (**d**) Schematic model of the evolution of EDDM and EDDML. The boxes represent the organization of EDDM and EDDML proteins whereby repeat units in the central domain are indicated by red boxes. *n*, number of central sequence repeats

Amino acid sequences, obtained by in silico translation of *EDDM* and *EDDML* coding sequences, were aligned to define common and divergent sequence features. EDDM and EDDML proteins contain 3 domains, all of which are characterized by high cysteine content. The amino- and carboxy-terminal domains are only partially conserved between birds and crocodilians whereas the central domain consists of at least 18 repeats of a sequence motif in both clades (Fig. 4a–c). Crocodilian EDDML proteins have 19–21 imperfect repeats of a 16-residue sequence and avian EDDM proteins have 18–54 imperfect repeats of a 15-residue sequence (Fig. 4b). In birds, the repeat sequence includes the DPCC motif, that is referred to in the protein name “EDDM.” The carboxy-terminal cysteine of this motif is not present in EDDML proteins of crocodilians, but cysteine is conserved at two other positions in birds and crocodilians (Fig. 4c). Another cysteine residue is present in the repeat of crocodilians, so that the average number of cysteine residues per repeat is 4 in all archosaurs. The sequence comparisons led to the evolutionary model depicted in Fig. 4d, which suggests that an EDDM-like gene originated in a common ancestor of archosaurs after the divergence from the turtle lineage, a central sequence motif underwent amplification in stem archosaurs, and further sequence changes both within and outside of the repeats led to divergent features of EDDM and EDDML in modern archosaurs. The number of central sequence repeats varied between the subclades of birds without an obvious correlation with an integumentary feature or lifestyle trait (Suppl. Fig. S4). Importantly, both the high cysteine content and the repetitive central domain are suggested to have emerged prior to the split of the avian and crocodilian lineages. Thus, our data point to an evolutionary origin of an epidermal differentiation protein with EDDM-like features in a common ancestor of birds and crocodilians, and this protein was subsequently co-opted for a new role as a component of feathers evolving in birds.

## Discussion

The present study extends previous investigations on avian epidermal differentiation by determining the expression pattern and evolution of a gene that encodes the cysteine-rich protein EDDM in the chicken. Our immunolocalization of EDDM in feather barbs and barbules contributes to ongoing characterization of the complex molecular architecture that makes feathers mechanically resistant, yet elastic skin appendages, and the results of comparative genomics provide new insights into the molecular evolution of feathers.

The high cysteine content of EDDM is comparable to that of avian EDCRP, another cysteine-rich protein component of feathers (Strasser et al. 2015), and mammalian cysteine-rich keratin-associated proteins (KRTAPs), which are components of hair and nails (Rogers et al. 2001; Deb-Choudhury 2018; Plowman 2018; Wu and Irwin 2018). While KRTAPs are encoded by genes outside of the EDC, the *EDCRP* gene is located in the EDC but within a different region than *EDDM* (Fig. 1). While the avian EDC segment localized between *S100A9*/*S100A12* and *Loricrin* lacks a counterpart in the mammalian EDC (Henry et al. 2012; Poterlowicz et al. 2017), the EDC segment containing *EDDM* in birds is syntenic with the human cluster of *Late cornified envelope* (*LCE*), *Cysteine-rich C-terminal 1* (*CRCT1*), and *Keratinocyte proline–rich protein* (*KPRP*) genes (Fig. 1). Among human EDC proteins, KPRP has the highest number of cysteine residues. KPRP was detected by proteomics, in human and mouse nails (Rice et al. 2010; Jaeger et al. 2019) and, by immunohistochemistry, also in the granular layer of human epidermis (Lee et al. 2005). Thus, proteins competent in the formation of disulfide bonds via multiple cysteine residues likely contribute to the mechanical and chemical resistance of cornified skin derivatives in diverse amniotes.

Our immunostainings detected EDDM in the cornifying cells of barb and barbules as well as in the embryonic subperiderm. This pattern is similar to that of feather CBPs (Sawyer et al. 2003), EDCRP (Strasser et al. 2015), and EDMTFH (Alibardi et al. 2016), and suggests common mechanisms of gene regulation for the concerted synthesis of these proteins. The most mature portions of feathers were immunonegative for EDDM and despite testing several conditions of protein extraction under reducing conditions, we could not detect EDDM by western blot analysis. Most likely heavy intermolecular cross-linking of EDDM to other structural proteins in differentiated cells of feathers prevents access to antibodies and extraction of EDDM for detection as a soluble protein. As EDDM was previously detected in a proteomic analysis of feathers, which involved proteolytic digestion and mass spectrometry of peptides (Rice et al. 2013; Strasser et al. 2014), the immunohistochemical detection of EDDM in feathers is supported by a mechanistically independent method.

The identification of EDDM orthologs in crocodilians, which represent the closest phylogenetic relatives of birds, and the detection of EDDM in the subperiderm of scutate scales of chicken embryos suggest that EDDM has not specifically evolved as a feather protein. The crocodilian orthologs of EDDM share many sequence features, including the repeat-rich central domain and the high cysteine content, with avian EDDM. Therefore, these features have most likely been inherited from the last common ancestor of birds and crocodilians which lived around 240 million years ago (Kumar et al. 2017) and according to current knowledge, did not have feathers. Interestingly, the evolutionary origin of another feather protein, EDCRP, could also be traced back to the last common ancestor of extant archosaurs (Holthaus et al. 2018b). Therefore, the co-option of epidermal differentiation for new roles as components of feathers appears to be an important theme, comparable to the co-option of claw keratins as structural proteins of hair in mammals (Eckhart et al. 2008).

Taken together, our data add EDDM to the catalog of feather proteins, also including feather CBPs (feather beta-keratins), EDCRP, and EDMTFH, which are encoded by single-coding exon genes within the EDC, also known as SEDC genes (Strasser et al. 2014). It is thus remarkable that the diversification of SEDC proteins within the EDC of archosaurs provided the molecular substrates for the evolution of feathers.

## Electronic supplementary material

ESM 1 (PDF 2169 kb)
