# Supplementary material for: Immunolocalization and phylogenetic profiling of the feather protein with the highest cysteine content
Source: Protoplasma. 2019 Apr 29;256(5):1257–65. doi: 10.1007/s00709-019-01381-3 (PMC6713690; doi:10.1007/s00709-019-01381-3)
Supplement: Supplementary file 1 — (PDF 2169 kb) [file 709_2019_1381_MOESM1_ESM.pdf]

Supplementary Table S1. Location of *EDDM* and *EDDML* genes and characteristics of encoded proteins

Species (common name)	Species (scientific name)	Gene	Accession number of genome sequence	CDS start	CDS end	Length of encoded protein (number of amino acid residues)	Number of cysteine residues	Cysteine content (%)
chicken	*Gallus gallus*	*EDDM*	NC_006112.4	2353285	2351312	657	152	23%
duck	*Anas platyrhynchos*	*EDDM*	NOIJ01000473.1	130734	132668	644	147	23%
pigeon	*Columbia livia*	*EDDM*	NW_004974408.1	129356	130906	516	118	23%
saker falcon	*Falco cherrug*	*EDDM*	NW_004994483.1	137875	135644	743	185	25%
Adélie penguin	*Pygoscelis adeliae*	*EDDM*	NW_008824664.1	164736	167225	829	191	23%
emperor penguin	*Aptenodytes forsteri*	*EDDM*	NW_008794623.1	148505	146016	829	208	25%
loon	*Gavia stellata*	*EDDM*	NW_009324734.1	48696	46741	651	156	24%
flycatcher	*Ficedula albicollis*	*EDDM*	NC_021696.1	412881	410173	902	218	24%
canary	*Serinus canaria*	*EDDM*	NW_007931203.1	408303	406717	528	117	22%
cuckoo roller	*Leptosomus discolor*	*EDDM*	NW_009873789.1	24669	26531	620	149	24%
lesser rhea	*Pterocnemia pennata*	*EDDM*	PTJI01000563.1	112626	111100	508	111	22%
great spotted kiwi	*Apteryx haastii*	*EDDM*	PTFD01000520.1	122833	120947	628	140	22%
ostrich	*Struthio camelus australis*	*EDDM*	NW_009271184.1	199388	200506	372	77	21%
American alligator	*Alligator mississippiensis*	*EDDML*	NW_017713819.1	255761	254523	397	84	21%
saltwater crocodile	*Crocodylus porosus*	*EDDML*	NW_017728904.1	721051	722289	412	86	21%
gharial	*Gavialis gangeticus*	*EDDML*	NW_017728960.1	3201932	3203158	408	85	21%

Note: CDS, coding sequence

>Gallus_gallus_EDDM
MLCQTDCQRGLPCLPPHVVLVRNLPVSRSVDPCNSVCSVSRLNTCADPCYYARVPQGTTTYLKLGSCDLRQIALDPCCLGITT
LTDPCCQDVTRCSSTRCVDPCCCKVTECSSTRYVDSCCQDATQCTTRCVDSCCQDATQCTTRCVDPCCQDVTQCTTRCVDPCR
QDATQCTTRCVDPCRQDATQCTTRCVDPCCQDVTKCTTRCVDPCRQDTTQCTTRCVDPCCQDVTKCTTRCIDPCRQDTTQCTT
RCVDPCCQDVTRCTTRCVDPCCKEVTRCTTRCVDPCCQDVTKCTTRCVDPCCKEVTKCTTTRCVDPCCKEVTRCTTRCADPCC
GEVTKCTTKYVDPCCRPVTRCATTCVDPCCGRVTKCTNKCVDPCYGAVTRCSTKCVEPCCEEVSKCTSRCVDPCCREVTKCTT
RCVDPCCGRVTKCARYKNPCCGEVSKCTTKCVDPCCGRVTKYVDPCCREVTSCKTRCVDPCCKEVTRCTTTCVDPCCAEVTRC
ATKCIDPCCQDATKCTTTCVDPCCREVTKCTTTCVDPCCQEVTKCTTTCVDPCCKEVTKCTTTCVDPCCQEVTKCTTTCVDPC
CREVAKCTTTCVDPCCQDATQCTTRCADPCCQDVTQCTSTCVDPCVPPCFVRPTPLCASICGRHYSISCADICCRK

>Pygoscelis_adeliae_EDDM
MYCQNDYQLRQLRLPPPTSVVKSFPLKRCVDPCDAPCNIQCLPPRVDPCCYVGIPQGTTTYVNLGNLGVTQPARCVDPCCRGV
TTCVPLCCGEVTTCVDPCCCKVTQCTTRCEDPCCEEAAKCTTTCVDPCCKEVTKCTTTCVDPCCKEVTKCTTRRVDPCCGEVT
KCTTTCVDPCCKEAAKCTTTCVDPCCGGVNECTTTCVDPCCKEVTKCTTRRVDPCCGEVTKCTTRCVDPCCGEVTKCTTRRVD
PCCGEVTKCTTTCVDPCGKEAAKCTTTYVDPCCKEAVKCTTTCVDPCGKEAAKCTTTYVDPCCKEAAKCTTTCVDPCGKEAAK
CTTTYVDPCCKEAVKCTTTCVDPCGKEAAKCTTTCVDPCCKEAAKCTTTCVDPCCKEAAKCTTTCVDPCCKEVTKCTTRRVDP
CCGEVTKCTTRRVDPCCGEVTKCTTRRVDPCCGEVTKCTTRCVDPCCGGVTKCSTRCVDPCCWEVTKCTTTCVDPCCKEVTKC
TTRRVDPCCGEVTKCTTRCVDPCCGEVTKCTTRCVDPCCGGVTKCTTRCVDPCCGEVTKCTTRRVDPCCGEVTKCTTRCVDPC
CWEVTKCTTRRVDPCCGEVTKCTTTCVDPCCGGVNECTTTCVDPCCKEVTKCTTTCVDPCCGGVNECTTTCVDPCCKEVTKCV
TTCVDPCCKEVTKCTTTCVDPCCKEVTKCTTTCVDPCCKEVTKCTTTCVDPCCKEVTKCTTTCVDPCCKEVTKCTTTCVEPCC
GGVTKCTTTCVDPCCKEVTKCTTTCVDPCCGGVQAVTKCVDSCPTACVTQAMPPCMGVCTSGCGQHYSITCADVCCRKCMAP

>Struthio_camelus_EDDM
MFCQNEYQYKQPCLPSAVFWLRSFPQQKCVESCGTVYNVQHLTQCMDPCCYSGVTQAATKYVDLCGNVGLTQSARCVDPCSCV
GPRYTTCVDPCCGGVTKCTTKYIDPCYGEVTKCTTKCVNPCCGEVTKCTTKCVDPCCGKATKCTTTCVDPCCEEVSKCTTKCV
DPCCREVTKCVSTCMDPCCSKLTKCATIFVDPCCEEVTKCTTTCVDPCCEEISKCTTRCVDPCCGAVTKCVTKCVDPCCSKVT
KCSTTCVDPCCEEVTKCTTTCVDPCCVAVTKCTTKCVDPCCGKATKCTTTCADPCCGEVTEHTNKCVDPCYRGVQAATKCVDS
CGTASVTQATSQCVDACTPACAHVYAISCADVCRRKCMAP

>Alligator_mississippiensis_EDDML
MAFPNQQQYKQPCLPPLVCIQKCPPRCVDQCDAACVKKHTDLHGNICAKSCTTKCVDSCDGISTMLCMTKCMDPCGAACVKEC
TTKCMCPSNTVCAKPCVTKYVDPCGTSCVMSCATPCLEPCNTICVKECVTKCMDPCGTFCAEPYVTKYGDPGCSNSAKPCITK
CVDSCNTVCVKECTTKCVDLCSTRYAKPCVTNCVDSCGTGCGKLCVTKCVDPCGTGCGKLCVTKCMDPCGTMCTKPCLTMCMN
PCSTRCAKPSVTKCVNLCGTMCGKPCITKHEDPCGTICVKECTTKCMDPCGIICTKPCVTKCVDPCTTSCVTSCVTKSTESCS
TVCIKKCTVKCVDTCSTICAKPFVPKCTDPCCPRCTASSGTTSMDPCAPVCKKTYPLQIVDLRLSKCPPVQQCCQKPKQC

>Crocodylus_porosus_EDDML
MAFPNQQQCKQPCLPSLVCIQKCPPGCVDQRDAACVKKHTDPCGNICTKSCTTKCVDSCNDISTMLCVTKCVDPCGAACVKEC
TTKCMCPSNTVCEKPCVTKYVDPCGTSCVTSCVTPCPEPGNTVCVKECITKCMDPCGTFCAEPYVTKCVDPSCSSSAKLCITK
CVDLCNTVCVKECTTKCVDPCSTRCAKPCVTNCVDLCGTVCAKPCITKCVDSCCTGCGKLCVTKYMDPCGTICAKPCLTMCMN
PCSTRCAKPTVTKCVELCSTVCPKPCIAKHGDPCGTICVKECTTKCMDPHDVICTKPCVTKCVDPCTTSCVTSCVTKCTESCN
TVCIKKCTVKCMDTCSTVCAKPFVPKCMDPCCPRCTASSGTMCMDPCAPVCKKTYPLQSVDPHLPKRPPVQQCCQKPKQC

>Gavialis_gangeticus_EDDML
MAFSNQEQCKQPCLPSLVCIQKSPPRCVDQCDAACVKKCTDPCGNICAKSCTTKCVDSCNGISTMLCMTKCVDPCGAACVKEC
TTKCMCPSNTVCAKPCVTKYVDPCGTSCVTSCVTPCPEPYNTVCVKECITKCMDPCGTFCAEPYVTKCVHPGCSSSAKLCITK
CVDSYNTVCVKECTIKCVDPCSTRCAKPCVTNCVDLCGTVCAKPCITKCVDSCCTGCGKQCVTKCMDPCGTICAKPCLTMCMN
PCSTRCAKPSVAKCVELCSTVCPKPCIAKHGDPCGTICVKECTTKCMDPRGVICTKPCVTKCVDPCATSCVTSCVTKCMESCS
TVCVKKCTVKCMDTVCAKPFVPKHMDPCCPRCMASGTMCMEPCAPLCKKTYPLQSVDPHLPKCPPVQQCCQKPKQG

Supplementary Figure S1. Amino acid sequences of representative EDDM and EDDML proteins. Cysteine residues (C) are highlighted by red fonts. The epitope of the anti-EDDM antibody used for immunohistochemistry is underlined. GenBank accession numbers of the genes encoding these proteins are shown in Supplementary Table S1.

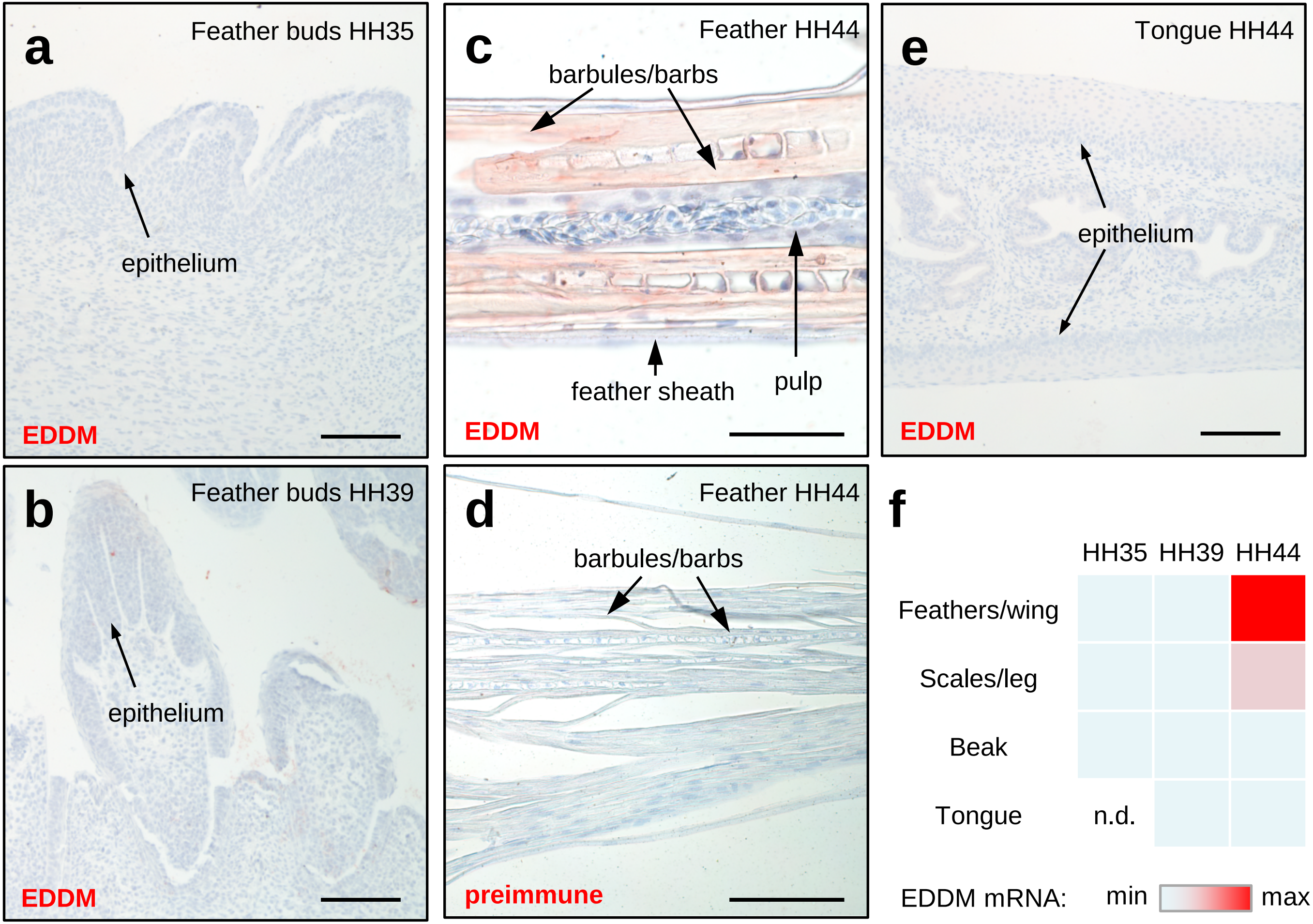

Supplementary Figure S2. Immunohistochemical and RT-PCR analysis of EDDM in chicken embryos. Expression of EDDM in chicken tissues was investigated by immunohistochemistry (**a**-**c**, **e**) and RT-PCR (**f**). Tissue sections from the indicated body sites and embryonic development stages (HH35, HH39, HH44) were subjected to immunohistochemical staining (red) with an antiserum against EDDM (**a**-**c**, **e**) or with preimmune serum (**d**). Nuclei were counterstained with hematoxylin (blue). Bars: 100 µm (**a**, **b**, **e**), 50 µm (**c**, **d**). (**f**) The abundance of EDDM mRNA, relative to a house-keeping gene, was determined by quantitative RT-PCR. The results are shown in a heatmap in which blue indicates low and red indicates high expression levels. n.d., not determined.

```
          1
  Chicken TGTCGCATGTACCACCTC--AGTGGCTAGGTTTCATTAAAAATTATAATGTAAGCAAGTA
   Pigeon TGTCACATATACCACCTC--AGTGGCTATGTTTCATTAAAA-TTATAATGTAAGCACAGA
  Ostrich TGTCATGTGTACCACCTC--AGTGGCCAGGTTTCATTAAAAATGATAATGTAAGCAAGTC
Alligator CCTCACAAGCTCCTTCTTGAATTGAGAGGTTTCCATTGAAAACCATGATGCAAACAAGCA
Crocodile CCTCACAAGCTCCTTCTTGAATTGAGAGGTTTCCATTGAAAACCATGATGCAAACAAGCA
  Gharial CCTCACAAGCTCCTTCTTGAATTGAGAGGTTTCCATTGAAAACCATGATGCAAACAAGCA
          ^^     ^ ^^  ^^  ^^^ ^  ^^ ^    ^    ^    ^   ^   ^  ^

          61
  Chicken ATTTTAGGAT-TGGAGGTGGAGCAGGTCTGGATTATT-AAAAAATTGTATTTCCATACCC
   Pigeon ATTTAATGAC-TGGAGGTGGAACAGGTCTGGATTATT-AAAAA-TTATATTTCCATACTC
  Ostrich ATTTAAGGAT-TGGAGGTAGAGCAGGTTTGACTTACT-AAAAATTT-TATTTCCATACCC
Alligator GTGTGAGGATCTGTAGGTGGAACCAGTTTAGATAGTTTAAAAAGTCATATTTTCATACTG
Crocodile CTGTAGGGATCTGTAGGTGAAACAGGTTTGGATAGTTAAAAAAGTCACATTTTCATACTG
  Gharial GTGTGCGGATCTGTAGGTGGAACAGGTTTGGATAGTTGAAAAAGTCACATTTTCATACTG
            ^       ^  ^                   ^^          ^      ^      ^

          121                                     TATA box
  Chicken CTGAGGTGTCTCACTTCCGGAGACAGGGACATTGAAATTGTATAAAAGGG--TTCATATT
   Pigeon CTGAGGTGTCTCACTTCCGGAGACAAGGACACTGAAATCATATAAAAGGA--TACATATT
  Ostrich CTGAGGTGTCTCACTTCCGGAGACACGGACACTAAAATCATATAAAAGGG--TTCATATT
Alligator CTGGGATGTCTCATTTCTGGACATGGGACCTCTGACTCCATCTAGAAGGGCTTGCACATC
Crocodile CTGGGATGCCTCGTTTCTGGACATGGGACATCTGACTCCATATAGAAGGGCTTGCACATC
  Gharial CTGGAACGCCTCATTTCTGGACATGGGACGGCTGACTCCATATAGAAGGGCTTGCACATC
             ^ ^       ^   ^   ^ ^^  ^       ^^^      ^     ^^ ^  ^  ^

          181                                                    intron
  Chicken GTAGTGTTTCCCAAGCCATCTTGGTTTCGTTAGCTTGTTCCTGGTGGTGAATCGGGTAAG
   Pigeon GTAGAGTTTCTCAAACCATTTCGGTTTCATTTGCTAGTTGCTGGTGGTGAACTGGGTAAG
  Ostrich GCAGTGTTTCTCAAACCATTTTGATTTCCTTTGCTTCTTCCTGTTAGAGATTTGGGTAAG
Alligator TCAATGCTCCTACAACCATTTC-------TTCACAAGGTTGCATTGGTGATTTGGGTAAG
Crocodile TCAATGCTCCTACAACCATTTC-------TTCACAAGGTCACATTGGTGATTTGGGTAAG
  Gharial TCAATGCTCCTACAACCATTCC-------TTCACAAGGTCGCATTGGCAATTTGGGTAAG
          ^  ^  ^ ^  ^^         ^ ^^^^    ^ ^  ^   ^^
```

Supplementary Figure S3. Alignment of nucleotide sequences of the proximal promoter, the non-coding exon 1, and the start of the intron of *EDDM* and *EDDML* genes. Canonical TATA box and intronic sequences are highlighted by yellow and grey shading, respectively. Red and blue letters indicate nucleotides conserved in 100% and >60% of the species, respectively. Positions where a nucleotide is conserved in all *EDDM* orthologs and a different nucleotide is conserved in all *EDDML* genes are marked with the symbol “^”.

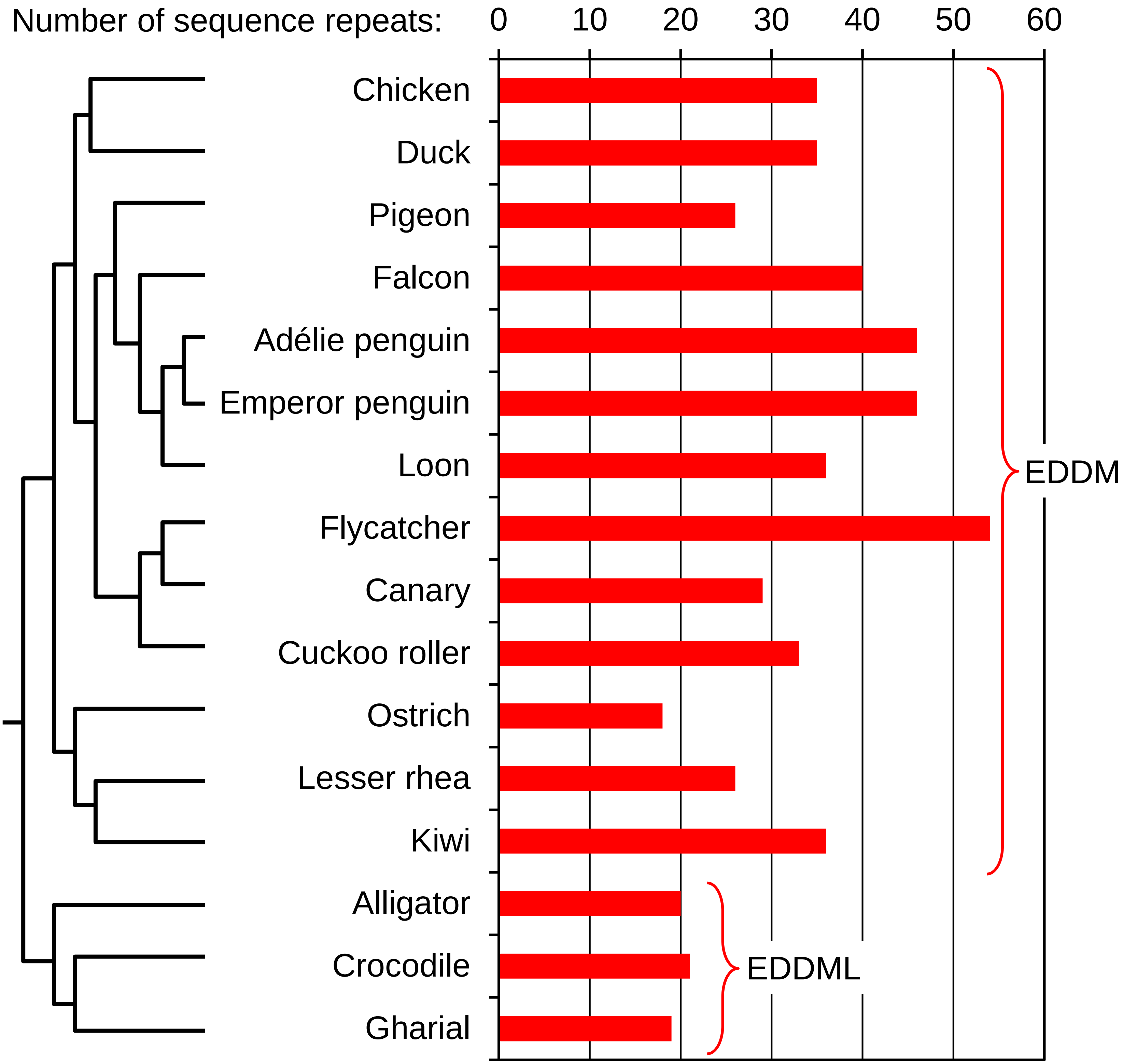

Supplementary Figure S4. Numbers of central sequence repeats of EDDM and EDDML in phylogenetically diverse birds and crocodilians. The numbers of EDDM and EDDML central sequence repeats, predicted from coding sequence of genes in Supplementary Table S1, were mapped onto a simplified phylogenetic tree of archosaurs. The phylogenetic tree is based on information from http://www.timetree.org (Kumar et al., 2017). EDDM, Epidermal Differentiation protein containing DPCC Motifs; EDDML, EDDM-like.
